# Vitamin D: Brain and Behavior

**DOI:** 10.1002/jbm4.10419

**Published:** 2020-10-18

**Authors:** Darryl Walter Eyles

**Affiliations:** ^1^ Queensland Centre for Mental Health Research The Park Centre for Mental Health Wacol Australia; ^2^ Queensland Brain Institute University of Queensland St. Lucia Queensland Australia

**Keywords:** BRAIN, DEVELOPMENT, VITAMIN D DEFICIENCY, NEUROPROTECTION

## Abstract

It has been 20 years since we first proposed vitamin D as a “possible” neurosteroid.^(^^1^^)^ Our work over the last two decades, particularly results from our cellular and animal models, has confirmed the numerous ways in which vitamin D differentiates the developing brain. As a result, vitamin D can now confidently take its place among all other steroids known to regulate brain development.^(^^2^^)^ Others have concentrated on the possible neuroprotective functions of vitamin D in adult brains. Here these data are integrated, and possible mechanisms outlined for the various roles vitamin D appears to play in both developing and mature brains and how such actions shape behavior. There is now also good evidence linking gestational and/or neonatal vitamin D deficiency with an increased risk of neurodevelopmental disorders, such as schizophrenia and autism, and adult vitamin D deficiency with certain degenerative conditions. In this mini‐review, the focus is on what we have learned over these past 20 years regarding the genomic and nongenomic actions of vitamin D in shaping brain development, neurophysiology, and behavior in animal models. © 2020 The Author. *JBMR Plus* published by Wiley Periodicals LLC on behalf of American Society for Bone and Mineral Research.

## Introduction

Readers of this, and other contributions to this issue will be aware of the wide range of nonskeletal targets for vitamin D. In particular, the last 20 years have been a fertile period for the investigation of vitamin D and its diverse functions in the brain. The distribution of the vitamin D receptor (VDR) and the enzyme associated with the synthesis of the active form of the hormone 1‐alpha hydroxylase (*CYP*27B1) have been mapped in human brain,^(^ ^3^ ^)^ along with studies showing the VDR is present in numerous brain cells such as oligodendrocytes, astrocytes, microglia, and neurons.^(^ ^2^, ^4^, ^5^ ^)^

Experimentally induced variations in vitamin D status have been shown to affect brain cell differentiation, neurotrophin expression, cytokine regulation, neurotransmitter synthesis, intracellular calcium signaling, antioxidant activity, and the expression of genes/proteins involved in neuronal structure, physiological function, and metabolism.^(^ ^6^, ^7^ ^)^ Therefore, perhaps it comes as no surprise that vitamin D status should be related to a number of clinical brain disorders. For more than a decade now inadequate levels of vitamin D have been linked with numerous adverse brain‐related outcomes. In particular, developmental vitamin D (DVD) deficiency has been linked with schizophrenia ^(^ ^8^, ^9^ ^)^ and more recently autism.^(^ ^10^, ^11^, ^12^ ^)^ Adult vitamin D (AVD) deficiency has also been linked with schizophrenia, Alzheimer disease (AD), dementias, and adult disorders of cognition (for a review, see Groves et al^(^ ^13^ ^)^). There are also strong links with Parkinson disease (PD).^(^ ^14^ ^)^

It is not our purpose here to concentrate on the clinical epidemiological literature. For detailed reviews of these associations, the reader is referred to two excellent recent summaries.^(^ ^13^, ^15^ ^)^ We will return to a discussion of the interpretation of this clinical literature at the end of article. Rather, the purpose here is to focus on the latest preclinical literature modeling these epidemiological links and discuss plausible biological mechanisms. Convincing evidence will be presented connecting vitamin D deficiency in animals with behavioral phenotypes of relevance to the aforementioned clinical conditions. The biological plausibility that low levels of vitamin D adversely affect brain development and function is now well‐established. Our task now is to discover exactly how low levels of vitamin D change the function of specific brain cells/circuits, predisposing an individual to develop such disorders and to see if correcting vitamin D status can ameliorate phenotype/symptom severity.

### *Dedication*

Like many of the other contributors to this special issue of *JBMR Plus*, I would like to honor Tony Normans’ legacy. When I went to my first Vitamin D workshop in Maastricht in 2003 I felt like a total imposter. What was a neuroscientist doing in an endocrine meeting where—to the best of my knowledge—no one had even mentioned the brain before? I acknowledge Tony for creating and sustaining this meeting, which, for me at least, has become a truly collegial environment for collaboration and for allowing us a continuing platform to communicate our research.

## Vitamin D Signaling and Metabolism in the Brain

The major circulatory form of vitamin D, 25‐hydroxyvitamin D_3_ [25(OH)D_3_], and its active hormonal form, 1,25‐hydroxyvitamin D_3_ [1,25(OH)_2_D_3_] are present in the brain.^(^ ^16^, ^17^ ^)^ Although the exact concentrations are debatable, they are likely to be much lower than those levels found in blood. Various technical issues in their extraction and method of quantification make claims of absolute amounts difficult at this time. These issues have been dealt with extensively elsewhere.^(^ ^15^ ^)^

Immunohistochemical evidence for the VDR is far stronger. The VDR has been confirmed in human, mouse, rat, chick, and zebrafish brains.^(^ ^3^, ^18^, ^19^, ^20^, ^21^, ^22^ ^)^ VDRs in brain are also functional, specifically binding to DNA response elements when liganded.^(^ ^23^ ^)^ In response to claims from some researchers that immunological detection of the VDR was open to errors,^(^ ^24^, ^25^ ^)^ we have provided unambiguous evidence via mass‐spectrophotometric protein sequencing of electrophoretically resolved proteins from adult rodent brains, which identified five unique VDR peptides with a CI >99%.^(^ ^26^ ^)^ The regional organization of VDR in human brain is remarkably consistent with that published for the rat. For example, VDR expression in both rat and human cerebellum is restricted to the granule cells and was completely absent from Purkinje cells.^(^ ^3^, ^19^ ^)^ In the human hippocampus, VDR immunoreactivity was strongest in CA1 and CA2 pyramidal cells with a marked reduction within CA3,^(^ ^3^ ^)^ a finding replicated in rats by two separate groups.^(^ ^19^, ^21^ ^)^ Within both the rat and human hypothalamus, the most densely labeled nuclei were the supraoptic and paraventricular nuclei.^(^ ^3^, ^18^ ^)^ VDR immunoreactivity was also completely absent in the large, presumably cholinergic neurons from the nucleus basalis in both rat and human brains.^(^ ^27^ ^)^ Finally, the concentration of VDR protein within the large dopaminergic neurons of the substantia nigra has now been confirmed in both rat and human brain.^(^ ^28^ ^)^ Importantly, VDR in brain is assumed to be functional in that it is able to specifically bind DNA response elements when bound to ligand.^(^ ^23^ ^)^ This close cross‐species overlap in VDR distribution validates the use of rodents in modeling vitamin D‐related brain outcomes.

The temporal nature of VDR expression in the developing brain has been qualitatively mapped in both rat^(^ ^29^ ^)^ and mouse brain.^(^ ^30^ ^)^ The immunohistochemical presence of VDR emerges on embryonic day (E) 12 (E12) in rats and E11.5 in mouse. As found in adult brain, there is a broad distribution across a variety of brain regions. In the developing rat brain, the VDR appeared to be localized in differentiating fields.^(^ ^29^ ^)^ This may be highly relevant given the role of vitamin D as a potent differentiation agent in a variety of cell types.^(^ ^31^ ^)^ We have provided more evidence that VDR signaling may be relevant to brain cell proliferation by identifying an intense VDR immunohistochemical presence in the ependymal surface of the lateral ventricles in neonatal rats.^(^ ^32^ ^)^ This is a site that represents the richest source of cell division in the postnatal brain.

Our initial studies of the time course for VDR protein and mRNA expression in the rat brain showed a general increase between E15 and E23.^(^ ^33^ ^)^ Since then, we have studied the ontogeny of the VDR in developing rodent brain at the immunohistochemical, mRNA, and protein levels. We confirmed earlier findings that the VDR is present at the early embryonic age of E12, but this staining did not appear to be cellular. By E15, clear punctate staining was obvious in the nucleus of dopaminergic neurons gaining a mature pan‐nuclear appearance by birth in these cells.^(^ ^28^ ^)^ This pattern of expression was largely confirmed at the mRNA and protein levels.^(^ ^28^ ^)^ We have confirmed VDR protein and mRNA were present in the neonatal brain in a subsequent study.^(^ ^26^ ^)^ The appearance of the VDR and its increasing expression in the embryo coincides with the onset of neuronal differentiation in the developing brain. Although a causal association cannot be directly established from these anatomical studies, they are consistent with vitamin D operating via its receptor to either directly or indirectly mediate features of neuronal apoptosis and cell cycle.

Finally, the enzyme that catalyzes conversion of 25(OH)D_3_ to the active or hormonal form of vitamin D, 1,25(OH)_2_D_3_, *CYP*27B1 is clearly present in the fetal human^(^ ^34^ ^)^ and adult brain,^(^ ^3^, ^35^ ^)^ suggesting local production of the active hormone may be possible in human brain. The enzyme was detected in the cytoplasm of both neurons and non‐neuronal cells.^(^ ^3^ ^)^ As with the VDR, the pattern of expression of this enzyme had regional and subregional specificity. The regions that stained the strongest were the supraoptic and paraventricular nuclei within the hypothalamus and the substantia nigra. Hydroxylation of 25(OH)D_3_ at position 24 by the enzyme *CYP*24A1 is a major catabolic pathway for vitamin D metabolites. There appears to be a selective distribution of *CYP*24A1 and *CYP*27B1 in non‐neuronal cells with *CYP*24A1 primarily found in astrocytes and *CYP*27B1 in microglia within brain.^(^ ^36^ ^)^

## Developmental Vitamin D Deficiency and Brain Development

The effects of manipulating vitamin D signaling on brain development have been measured by either genetically ablating the VDR or *CYP*27B1, or in models of dietary deficiency. Here we will not discuss brain functional data from the genetic models further as the physiology of these animals is often compromised, thus distorting behavior (see the section below). However, there is abundant research from models of dietary restriction. Our group was the first to create a dietary model of developmental vitamin D (DVD) deficiency in rodents specifically to examine developmental brain‐related outcomes.^(^ ^37^, ^38^, ^39^ ^)^ These vitamin D‐deficient dams and DVD‐deficient offspring have normal calcium and phosphate levels: Neither the dams nor their offspring have a rickets‐like phenotype.^(^ ^40^ ^)^ Because we developed this model in rats in 2003 it has been adapted in both other rat or mouse strains to examine the long‐term neuropsychiatric outcomes of DVD deficiency.^(^ ^41^, ^42^, ^43^, ^44^ ^)^ In summarizing findings from such models, they would all appear to produce changes in brain cell differentiation, anatomy, neurotransmitter production, and gene and protein expression.

DVD‐deficient rat embryos have increased brain cell proliferation,^(^ ^32^, ^38^, ^45^ ^)^ and reduced apoptosis,^38^, ^44^, ^45^ ^)^ along with corresponding changes in cell‐cycle and apoptotic gene expression. These findings in DVD‐deficient embryonic brains are in accord with in vitro studies in brain cells.^(^ ^46^, ^47^ ^)^ When the anatomy of DVD‐deficient embryonic brains was examined, it was revealed that the newborn offspring of DVD‐deficient rats had slightly larger brains. This corresponded with an increased volume of the lateral ventricles and a smaller neocortical width.^(^ ^38^ ^)^ The structural brain phenotype of embryonic DVD‐deficient mice, however, would appear to be quite different, with lateral ventricles being shown to be reduced in the embryonic mouse brain.^(^ ^44^ ^)^ This was also accompanied by reduced brain length,^(44^ ^)^ rather than longer brains as seen in DVD‐deficient newborn rats.^(^ ^38^ ^)^ Hippocampal volumes were also shown to be reduced in the DVD‐deficient mouse, but only in female newborns.^(^ ^48^ ^)^ Many of these anatomical differences persist into adulthood. The enlarged lateral ventricles seen in DVD‐deficient rat neonates^(^ ^38^ ^)^ persist into adulthood if vitamin D deficiency is continued until weaning.^(^ ^49^ ^)^ The opposite finding regarding a decrease in lateral ventricles in mouse embryos also appears to persist into adulthood.^(^ ^48^, ^50^ ^)^ Two separate groups have also now shown that DVD‐deficiency in C57B6J mice produces a larger striatum and smaller hippocampus in adult males.^(^ ^48^, ^50^ ^)^ The reason for why DVD deficiency would induce contrasting brain structural findings between species remains unknown. There may be a differential effect of DVD deficiency on brain cell proliferation between rats and mice, but until this is directly studied in mice this remains speculative. Vitamin D deficiency has also been associated with a 28% increase in lateral ventricles in aged humans.^(^ ^51^ ^)^

Of all neurotransmitters to be linked with DVD deficiency, dopamine (DA) is the one most reported. DVD deficiency may also adversely affect the ontogeny of other neurotransmitter systems such as serotonin; however, as far as we are aware, such evidence remains only at the in vitro level.^(^ ^52^, ^53^, ^54^ ^)^ As previously mentioned in developing brains, the VDR appears very early, at E12.^(^ ^28^, ^29^ ^)^ This represents the peak age when most DA neurons are being born.^(^ ^55^ ^)^ When mesencephalon was harvested from vitamin D‐deficient embryos at this age, we showed DVD‐deficient embryonic brains had a reduction in Nurr 1 and p57kip2a, which are two crucial specification factors for the maturation of DA neurons.^(^ ^56^ ^)^ Genetically ablating these two factors leads to a reduction in DA cell number and altered positioning.^(^ ^57^, ^58^, ^59^ ^)^ In a later study, we confirmed that early positioning of DA neurons in the developing mesencephalon was indeed altered with an increase in laterally migrating DA neurons in DVD‐deficient brains.^(^ ^60^ ^)^ We also measured DA levels in DVD‐deficient neonatal forebrain, and found that, although DA levels were normal, its metabolism was altered with increased ratios of the two major DA metabolites, 3,4‐dihydroxyphenylacetic acid (DOPAC) and homovanilic acid (HVA).^(^ ^61^ ^)^ This was accompanied by a reduction in catechol‐O‐methyltransferase (COMT), the enzyme that converts DOPAC to HVA.^(^ ^61^ ^)^ Additionally, a recent study has shown that gene expression and protein content of the rate‐limiting enzyme in DA production, tyrosine hydroxylase (TH) is reduced in DVD‐deficient fetal mouse brains.^(^ ^44^ ^)^ This same study has shown DVD deficiency in mice decreases the neurotrophin brain‐derived neurotrophic factor (BDNF) at early stages of brain development with a reversal at later stages.^(^ ^44^ ^)^ This study also showed DVD deficiency decreased the expression of TGF‐β1, an important factor in dopaminergic differentiation. Enzymes involved in corticosterone metabolism were also shown to be decreased.^(^ ^62^ ^)^ Some DA abnormalities persist through to adulthood with DA transporter density in the caudate putamen and DA binding affinity in the nucleus accumbens both being increased in DVD‐deficient adult rats.^(^ ^63^ ^)^

DVD deficiency also has long‐term effects on gene and protein expression in adult brains. Gene array analysis of whole brain and proteomics in the prefrontal cortex and hippocampus of adult animals who were subjected to DVD deficiency show alterations in the expression of 74 genes and 36 proteins involved in such diverse functions as cytoskeleton maintenance, calcium homeostasis, synaptic plasticity and neurotransmission, oxidative phosphorylation, redox balance, protein transport, chaperoning, cell cycle control, and posttranslational modifications.^(^ ^64^, ^65^ ^)^ A study of protein expression in the nucleus accumbens of DVD‐deficient rats showed that although the degree of gene dysregulation was mild, there were significant alterations in several proteins involved in either calcium binding (calbindin, calretinin, and hippocalcin) or mitochondrial function.^(^ ^66^ ^)^ One earlier study also showed DVD deficiency induced reductions in NGF, and neurofilament proteins indicative of delayed neuronal maturation.^(^ ^49^ ^)^

## DVD Deficiency and Animal Behavior

### Behavior of DVD‐deficient rats

Alterations in maternal/pup interactions can produce long‐lasting changes in offspring behavior.^(^ ^67^ ^)^ In particular, the quality of nursing behaviors, pup/dam communication via pup ultrasonic vocalizations and how the dam retrieves pups once separated from the main nest can all produce long‐term effects in adult behavior. Pup/dam interactions have recently been examined in DVD‐deficient animals. Yates and coworkers showed vitamin D‐deficient dams exhibited decreased licking and grooming of their pups, but no differences in pup retrieval. Perhaps consistent with this, DVD‐deficient pup ultrasonic vocalizations (a form of pup/dam communication) was also increased. As adults, males that had been exposed to vitamin D deficiency in early life exhibited decreased social behavior, impaired learning and memory outcomes, and increased grooming behavior.^(^ ^68^ ^)^ We have also examined many of these interactions in DVD‐deficient rats. Again, we found an increase in pup vocalizations,^(69^ ^)^ and similar reductions in social interaction in offspring at later stages of development. We also found some early climbing and self‐righting reflex deficits indicative of delayed development.^(^ ^69^ ^)^ Additionally, these animals had impairments in normal ethologically valid stereotyped digging behavior. Many of these behaviors are considered important phenotypes in animal models of relevance to autism.^(^ ^70^ ^)^

As adults, locomotion in response to a novel open field is enhanced in DVD‐deficient rats.^(^ ^37^ ^)^ Locomotion in response to psychomimetic agents has also been assessed in DVD‐deficient rats. Using the N‐methyl‐D‐aspartic acid receptor antagonist, MK‐801, an agent well‐known to induce hyperlocomotion, adult male DVD‐deficient rats have been repeatedly shown to have enhanced locomotor activity compared with controls.^(^ ^40^, ^71^, ^72^ ^)^ This MK‐801‐induced hyperlocomotion in adult male and female DVD‐deficient rats is associated with a significant reduction in MK‐801 binding in the caudate putamen.^(^ ^72^ ^)^ We have also found that the later period of gestation was the most‐relevant period of DVD deficiency for this effect.^(^ ^40^ ^)^ This is reminiscent of our earlier findings regarding structural changes in the brains of these animals.^(^ ^49^ ^)^ Therefore, it appears the developmental window in which vitamin D deficiency is induced is also critical for behavioral outcomes.

DVD‐deficient rats were also selectively sensitive to the locomotor‐enhancing effects of amphetamine, a drug that induces presynaptic DA release.^(^ ^63^ ^)^ A number of studies have also shown that DVD‐deficient rats are selectively sensitive to postsynaptic DA blockade, in particular the DA 2 receptor blocker, haloperidol (which is a widely used antipsychotic agent). The locomotor retarding effects of haloperidol appeared to be greater in DVD‐deficient animals when hyperlocomotion had first been induced by MK‐801.^(^ ^71^ ^)^ In a separate study, haloperidol was shown to normalize an endogenous habituation deficit in DVD‐deficient animals whereas it resulted in habituation deficits if administered to control animals.^(^ ^42^ ^)^ Using electrophysiological recordings from the hippocampus of freely moving rats, a subsequent study investigated long‐term potentiation (LTP), which is a cellular correlate of learning and memory.^(^ ^73^ ^)^ It was shown that DVD‐deficient rats had enhanced LTP, and this was reversed by treatment with haloperidol. DVD‐deficient rats also appeared to have normal prepulse inhibition^(^ ^71^ ^)^ and working memory, but disrupted latent inhibition, which is a measure of attentional processing.^(^ ^74^ ^)^ Although manipulating striatal DA release can affect all of these three behaviors, this potential mechanism has not yet been investigated in vivo.

In a continuous performance task developed by Turner and colleagues, DVD deficiency produced animals that had increased premature responding, reflecting increased impulsivity, and increased responding to nontarget stimuli. Both behaviors indicate a lack of response inhibition.^(^ ^75^ ^)^ Importantly, both of these behaviors were normalized with acute treatment with the antipsychotic clozapine.^(^ ^75^ ^)^ Finally, we have recently shown associative learning to be impaired in DVD‐deficient rats.^(^ ^76^ ^)^ Clearly, there are multiple behavioral abnormalities in these animals—many of which are of potential relevance to phenotypes of interest in schizophrenia and autism research.

### Behavior of DVD‐deficient mice

DVD deficiency in one mouse strain,129/SvJ, produced spontaneous hyperlocomotion in the open‐field arena, similar to DVD‐deficient rats.^(^ ^71^ ^)^ Another widely used strain, C57BL/6J mice exposed to DVD deficiency showed increased perseverative responses to the target stimuli in a continuous performance task.^(^ ^77^ ^)^ This indicates DVD deficiency induces similar deficits in response inhibition in both species. However, all other behavioral phenotypes of DVD‐deficient mice are distinctly different from the rat.

DVD‐deficient mice from both strains demonstrate an increased frequency of head dips in a hole board arena, indicative of increased exploratory behavior.^(^ ^78^ ^)^ This is in contrast to findings from DVD‐deficient rats on the same test.^(^ ^42^ ^)^ Also, the robust locomotor response seen in DVD‐deficient rats when exposed to MK‐801 or amphetamine is not found in mice.^(^ ^48^ ^)^ Another group has tested DVD‐deficient C57BL/6J mice on a hippocampal‐dependent memory task known as the olfactory tubing maze. A learning deficit was seen on the final day of training, with DVD‐deficient mice showing a reduction in the number of correct responses when compared with controls.^(^ ^50^ ^)^

Clearly, the behavioral phenotype of the DVD‐deficient rat is distinctly different to that of the mouse. However, the array of behaviors examined indicates subtle alterations in learning and memory in both species. The effects of DVD deficiency on cognitive function in children are far from clear. One study has shown children who were vitamin D deficient during pregnancy had delayed cognitive development,^(^ ^79^ ^)^ but this finding was not replicated in a larger study when a broader array of cognitive outcomes was assessed.^(^ ^80^ ^)^ Although a comprehensive summary of the differences in brain structural and behavioral outcomes in DVD‐deficient rats and mice has recently been published,^(^ ^81^ ^)^ an exhaustive comparison between the effects of DVD deficiency in both species has not yet been conducted. Perhaps a more useful line of inquiry would be an examination of which critical developmental window of exposure and which critical threshold of vitamin D deficiency are required to change brain function in adult offspring.

To induce vitamin D‐deficient signaling via a genetic approach, groups have either ablated the receptor^(^ ^30^, ^82^, ^83^, ^84^ ^)^ or the major synthetic enzyme for 1,25(OH)_2_D_3_ (*CYP*27B1).^(^ ^85^, ^86^ ^)^ In these models, the VDR or enzyme are constitutively ablated. Although VDR KO mice have impairments on a range of behaviors or relevance to psychiatry such as anxiety,^(^ ^87^ ^)^ neophobia,^(^ ^88^ ^)^ and altered nest building,^(^ ^89^ ^)^ they also have severe phenotypes unrelated to brain such as hypertension and increased fluid intake,^(^ ^90^ ^)^ cardiac hypertrophy,^(^ ^91^ ^)^ altered heart function,^(^ ^92^ ^)^ impaired energy metabolism,^(^ ^93^ ^)^ musculoskeletal changes,^(^ ^94^ ^)^ growth retardation, impaired motor coordination, and muscle fatigue.^(^ ^95^, ^96^ ^)^

To date, there are no published studies using conditional or brain‐specific VDR mutant mice to test the effects of transient or regional receptor disruption on brain development. Although tissue specific inactivation of *CYP*27B1 has been demonstrated,^(^ ^97^ ^)^ these homozygous mutants also have a confounding rickets‐like phenotype. These “off‐target” deficits complicate the interpretation of the genetic contribution of vitamin D signaling to behavior.

## Adult Vitamin D Deficiency/Supplementation and Brain and Behavior

As previously mentioned, there are also numerous studies linking low vitamin D status with schizophrenia, AD, dementias, and PD.^(^ ^13^ ^)^ To investigate the biological nature of these links, various preclinical models of adult vitamin D (AVD) deficiency have been developed. One complicating factor in many earlier animal studies of AVD deficiency was the failure to address hypocalcemia, which can radically affect brain function; therefore, such studies will not be discussed further here.

As cognition is impaired in most of the afore‐mentioned disorders linked with AVD deficiency, this behavior has been the most commonly assessed in AVD models; however, the picture is far from clear. Six weeks of vitamin D deficiency is insufficient to change cognitive responses in an AVD rat; but it did lead to premature responses indicating some effect on vigilance.^(^ ^98^ ^)^ There were also small changes in striatal neurotransmitter content including increased gamma‐aminobutyric acid (GABA) and alterations in DA turnover. Although much longer periods of vitamin D deficiency (6 to 12 months) increased reactive oxygen production in the brain,^(^ ^99^ ^)^ it also did not affect cognition.^(^ ^100^ ^)^ Mild cognitive deficits have been shown in some studies using AVD‐deficient mice,^(^ ^101^ ^)^ along with alterations in the major excitatory neurotransmitter in the brain, glutamate, and the major inhibitory transmitter GABA.^(^ ^102^ ^)^ How these alterations could directly relate to impaired cognition, however, is not immediately obvious. The addition of high‐dose cholecalciferol (10 times normal dietary levels) did appear to increase memory outcomes in one study.^(^ ^103^ ^)^

In related studies, AVD deficiency has been shown to increase corticosterone response to stressful events and increased avoidance times.^(^ ^104^ ^)^ One specific learning task did appear to be affected by AVD deficiency with AVD‐deficient rats having impairments in aversive spatial learning. Importantly, these findings were correlated with connectivity abnormalities in the major brain region associated with spatial navigation, the hippocampus.^(^ ^105^ ^)^

With respect to AD, there are numerous transgenic models mimicking the brain pathology of the disease. Given the ongoing reports of vitamin D deficiency in patients with AD, a number of studies have been initiated to see if cognitive decline and or brain toxicity could be attenuated by vitamin D treatment in such models. These studies have mostly supported the idea that vitamin D is neuroprotective. For instance, vitamin D‐enriched foods decrease brain pathology and prevent cognitive decline.^(^ ^106^ ^)^ These findings were largely replicated by dietary supplementation.^(^ ^107^ ^)^ Similar outcomes were found using acute exposure to 1,25(OH)_2_D_3,_^(^ ^108^ ^)^ with the hormonal form of vitamin D also appearing to increase elimination of pathological ß‐amyloid proteins from the brain.^(^ ^109^, ^110^ ^)^ Models of dietary insufficiency have also been shown to lead to greater AD‐related brain pathology.^(^ ^107^, ^111^ ^)^

Similar to the situation of AD, there are numerous genetic or toxin‐based models of PD. Again disease‐specific pathology would appear to be alleviated by acute administration of the active hormone, 1,25(OH)_2_D_3._^(^ ^112^, ^113^, ^114^, ^115^ ^)^ This would appear to be via upregulation of the rate‐limiting enzyme for DA production, TH.^(^ ^116^, ^117^ ^)^ We have now outlined the direct mechanisms for how vitamin D regulates TH gene expression (and see below).^(^ ^117^, ^118^ ^)^ 1,25(OH)_2_D_3_ also ameliorates the oxidative burden many of these PD models induce in the brain.^(^ ^119^ ^)^

## Vitamin D Regulates Essential Processes in Normal Brain Development and Function and Is Neuroprotective in Adult Brains

So far, we have outlined how vitamin D deficiency may adversely affect essential normal processes in brain development, adult brain function, and behavior. In this next section, we review the basic evidence for how vitamin D exerts influence over crucial events in brain ontogeny, such as axonal elongation, neurotrophin production, and neurotransmitter synthesis, as well as how it can act to protect neurons from a range of adverse exposures.

### Vitamin D and axonal growth

We were the first group to show the addition of 1,25(OH)_2_D_3_ to embryonic hippocampal explant cultures increased neurite outgrowth.^(^ ^46^ ^)^ This finding was replicated more recently in individual hippocampal neurons using the same concentration of 1,25(OH)_2_D_3._^(^ ^47^ ^)^ Both groups described a small, but significant elevation in NGF and assumed this effect was causal. Another group chose to examine the ability of ergocalciferol (vitamin D_2_) to enhance axon regeneration after peripheral denervation. These authors chose ergocalciferol based on an older study that claimed this was more potent than cholecalciferol in elevating 25OHD_2_ levels in rats.^(^ ^120^ ^)^ These authors were able to demonstrate increased axogenesis, axon diameter, and higher functional recovery if ergocalciferol treatment was initiated immediately after lesioning.^(^ ^121^ ^)^ We also now have new unpublished data replicating the neurite promoting potential of 1,25(OH)_2_D_3_ in developing DA neurons differentiated from (i) a neuroblastoma cell line, (ii) primary mesencephalic DA neurons, and (iii) explant mesencephalic cultures. We conclude that like all other neurosteroids, vitamin D enhances neurite extension. We are now exploring the molecular mechanisms for these effects.

### Vitamin D and neurotrophic factors

Vitamin D's actions in promoting neurotrophic factors, such as NT‐3, NT‐4,^(^ ^122^ ^)^ and nerve growth factor (NGF) in particular,^(^ ^46^, ^47^, ^122^, ^123^, ^124^ ^)^ have been well‐described. NGF is particularly important in the development and survival of hippocampal neurons in either cultured explants ^(^ ^46^ ^)^ or in individual cultured cortical neurons.^(^ ^125^ ^)^ Silencing VDR expression leads to a corresponding reduction in NGF production in primary cortical neurons.^(^ ^126^ ^)^ Administration of 1,25(OH)_2_D_3_ directly into the hippocampus of adult rats also induces NGF expression. Therefore, the evidence that vitamin D may be required for ongoing neuronal survival in adult brains via such mechanisms appears strong.^(^ ^127^ ^)^

Given its role in dopaminergic neuron differentiation and survival, there has also been a strong focus on vitamin D and neurotrophic factors specific to dopaminergic neurons such as glial‐derived neurotrophic factor (GDNF)^(^ ^128^, ^129^ ^)^ and BDNF again for its broad trophic actions in the developing and adult brain. Neural stem cells treated with 1,25(OH)_2_D_3_ show increased expression of NT‐3, BDNF, and GDNF.^(^ ^130^ ^)^ Cultured mesencephalic neurons, which contain most of the developing DA neurons in the brain, increase GDNF expression after 1,25(OH)_2_D_3_ administration with an increase in DA cell number also. This vitamin D‐mediated increase is blocked when GDNF synthesis is chemically blocked.^(^ ^131^ ^)^ We have recently shown direct genomic evidence for how vitamin D directly regulates the transcription of both receptors for GDNF. 1,25(OH)_2_D_3_ suppresses GDNF family receptor α 1, but the liganded VDR directly binds to the promoter of the proto‐oncogene tyrosine–protein kinase receptor Ret (C‐Ret), which is the other major receptor for this neurotrophin, to upregulate C‐Ret expression. Correspondingly, the maternal absence of vitamin D decreases C‐Ret expression in the developing rat mesencephalon.^(^ ^118^ ^)^

Very few studies to date have examined the effect of vitamin D on these neurotrophic factors in the developing brain. In the studies that have, one showed DVD deficiency in rats induced deficits in neonatal whole‐brain NGF and GDNF protein.^(^ ^38^ ^)^ A much more recent study also showed early reductions in BDNF and transforming growth factor‐β1 in DVD‐deficient embryonic mouse brains.^(^ ^44^ ^)^

Almost universally, the breadth of work over the past two decades indicates 1,25(OH)_2_D_3_ *increases*, and the absence of vitamin D in the maternal diet *reduces* the expression of these important neurotrophic factors in neurons and glia of developing brains. These factors remain highly attractive candidate pathways in understanding the role vitamin D plays in brain ontogeny.

### Vitamin D as a regulator of dopamine in development

1,25(OH)_2_D_3_ has been shown to alter cholinergic, dopaminergic, and noradrenergic neurotransmitter systems in vitro.^(^ ^132^, ^133^ ^)^ Our data to date on DVD‐deficient brains strongly and consistently indicate the absence of this vitamin during fetal brain development appears to produce adverse effects on developing DA systems—and to a lesser extent— on adult DA systems. We were the first group to report intense immunohistochemical staining of the VDR within TH‐positive neurons within the human substantia nigra.^(^ ^3^ ^)^ Since then, we have confirmed that TH‐positive neurons in the neuromelanin containing human nigra are VDR‐positive, and we have now mapped the ontogeny of VDR expression in rat brain.^(^ ^28^ ^)^ As previously discussed, the absence of vitamin D during development decreases the expression of crucial specification factors for DA neurons ^(^ ^56^ ^)^ and reduces enzymes involved in DA turnover with accompanying alterations in DA metabolites.^(^ ^61^ ^)^ We have recently shown DVD deficiency alters the positioning of the two major DA neuron clusters in embryonic brains with an imbalance between DA neurons in the substantia nigra compared with the ventral tegmentum, along with reductions in the expression of Nurr1 and TH indicative of a delay in DA neuron differentiation.^(^ ^60^ ^)^ In a latter study, we showed 1,25(OH)_2_D_3_ was capable of rescuing deficits in DA‐specification‐factor expression, DA progenitor cell number, and positioning abnormalities in DA neurons induced by maternal immune activation.^(^ ^134^ ^)^

We have confirmed that 1,25(OH)_2_D_3_ positively regulates TH mRNA and protein, and the metabolic product of TH, DA using a VDR‐overexpressing neuroblastoma cell system.^(^ ^117^ ^)^ Simply increasing VDR expression alone in the absence of 1,25(OH)_2_D_3_ is also sufficient to drive undifferentiated cells down a dopaminergic lineage.^(^ ^135^ ^)^ In addition, we have established that 1,25(OH)_2_D_3_ increases VDR regulation of a major metabolic enzyme for DA in the brain, COMT. Chromatin immunoprecipitation data confirm the liganded VDR binds to the COMT promoter, strongly suggesting a direct regulation of COMT gene expression.^(^ ^135^ ^)^ Another group has shown 1,25(OH)_2_D_3_ may drive TH and therefore DA production via a GDNF‐mediated mechanism.^(^ ^131^ ^)^ We and others are now engaged in trying to understand how such early changes in the formation of dopaminergic systems could affect downstream brain function in mature animals.^(^ ^136^ ^)^ 1,25(OH)_2_D_3_ has also been administered to newborn rats, and DA and noradrenalin measured in a variety of brain regions in these animals as adults. The authors found that DA and noradrenalin were elevated mainly in the brainstem of these animals as adults.^(^ ^137^ ^)^

Considered in its totality, the consistent findings of impaired DA neuron maturation in DVD‐deficient embryonic brains, impairments in behavior influenced by DA in DVD‐deficient adults, coupled with our most recent data showing vitamin D signaling in cultured neurons drives neuron maturation down a dopaminergic lineage, all combine to strongly suggest vitamin D plays a crucial role in the early ontogeny of DA systems. Given we have established that DVD deficiency is a developmental epidemiological risk factor for schizophrenia,^(^ ^8^, ^9^ ^)^ and that DA abnormalities are also strongly linked with this disease, these data may prove important for the etiology of mental illness.

### Vitamin D and possible neuroprotective mechanisms in the developing and adult brain

Here we focus on three common exposures: excessive calcium, ROS, and corticosterone, which are all naturally occurring in the brain, but when elevated experimentally and by analogy, in various disease states, are known to adversely affect brain function and in some cases lead to long‐term pathology. These exposures are all exacerbated by vitamin D deficiency and ameliorated by the addition of 1,25(OH)_2_D_3_ or dietary cholecalciferol.

Calcium transients are essential for normal neuronal function; however, unbuffered calcium is neurotoxic for brain cells. It is well‐known how vitamin D regulates calcium uptake in non‐neuronal cells, such as osteoblasts and osteosarcoma cells via direct regulation of calcium channels.^(^ ^138^, ^139^ ^)^ However, now the actions of vitamin D are being studied in neurons and brain. Studies in vitro show 1,25(OH)_2_D_3_ blocks calcium influx and therefore toxicity in cultured mesencephalic neurons^(^ ^140^ ^)^ or hippocampal neurons^(^ ^141^, ^142^ ^)^ via the downregulation of L‐type voltage‐sensitive calcium channels.^(^ ^126^ ^)^ Silencing VDR expression blocks this.^(^ ^126^ ^)^ However, the rapid nongenomic actions of vitamin D produce the opposite effect with an increase in calcium influx in cortical slices, which is again dependent on L‐type calcium channel activity.^(^ ^143^ ^)^ Recently, we have used calcium imaging, electrophysiology and molecular biology to further explore the nongenomic actions of 1,25(OH)_2_D_3_ on cortical neurons. We show physiological concentrations of 1,25(OH)_2_D_3_ rapidly enhance calcium influx, but only in a small subset of neurons. Somatic nucleated patch recordings revealed a rapid, 1,25(OH)_2_D_3_‐evoked increase in high‐voltage‐activated calcium currents, and again these were mediated by L‐type voltage‐gated calcium channels.^(^ ^144^ ^)^ Examination of the function of vitamin D signaling on the activity of these channels is worth further scrutiny, given the close links between genetic variants in these channels and schizophrenia.^(^ ^145^ ^)^

The antioxidant potential of vitamin D in a variety of tissues, including isolated neurons^(^ ^140^, ^146^ ^)^ and brain,^(^ ^114^, ^147^ ^)^ has long been known. In general, this is believed to be because of the ability of vitamin D to increase potent antioxidant molecules such as glutathione and cytochrome c. A recent study showed AVD deficiency increased ROS and extracellular calcium in the brain. This occurred along with impairments in GABA and glutamate release. Importantly, all deficits were normalized by reintroducing dietary vitamin D.^(^ ^148^ ^)^

One long‐speculated neuroprotective mechanism of 1,25(OH)_2_D_3_ in the brain has been the potential inhibition of nitric oxide (NO) production.^(^ ^4^ ^)^ In the brain, microglia are the immunologically responsive cells responsible for the production of inflammatory regulators such as NO. Early reports suggested vitamin D could affect neuroinflammation and microglial activation. 1,25(OH)_2_D_3_ inhibits the expression of inducible NO synthetase in the rat brain during either experimental allergic encephalomyelitis^(^ ^149^ ^)^ or after intracranial injection of LPS.^(^ ^150^ ^)^ 1,25(OH)_2_D_3_ also reduces the production of proinflammatory cytokines and NO in microglial cells.^(^ ^151^ ^)^ Later studies have focused on potential molecular mechanisms. Microglia when activated with LPS increase production of *CYP*27B1 and as a result, 1,25(OH)_2_D_3_. In an important study, when LPS‐induced elevation of NO was examined in cultured microglia, the addition of 25(OH)D_3_ reduced NO production presumably via local synthesis of the active hormone 1,25(OH)_2_D_3_. Confirmation of this came from treating these same microglia with silencing RNA directed against *CYP*27B1, which reversed the inhibitory effect of 25(OH)D_3_.^(^ ^152^ ^)^ Treatment with 1,25(OH)_2_D_3_ also attenuates LPS‐induced ROS production, NO accumulation, and inducible NO synthase expression in concentration‐dependent manners in primary cortical neurons in culture.^(^ ^153^ ^)^ Finally, a recent study in synaptosome preparations from vitamin D‐deficient adult rats showed increased ROS and higher calcium influx, indicating increased excitability. Importantly, this was reversed in rats in which vitamin D deficiency had been corrected by supplementation.^(^ ^148^ ^)^ In summary, microglia appear to represent the site for the antioxidant actions of vitamin D in the brain.

The secretion of glucocorticoids is a classic endocrine response to stress. Prolonged exposure to increased levels of this hormone induces neuronal atrophy and eventually cell death.^(^ ^154^ ^)^ In general, the cellular effects of 1,25(OH)_2_D_3_ and glucocorticoids are considered to be antagonistic.^(^ ^155^, ^156^, ^157^, ^158^, ^159^ ^)^ This would also appear to be the case in the brain: with 1,25(OH)_2_D_3_ antagonizing the effects of the corticosterone agonist dexamethasone on hippocampal neuron differentiation and glucocorticoid receptor function.^(^ ^160^ ^)^ This process is reversible with dexamethasone shown to decrease the expression of enzymes involved in both the synthesis and turnover of 1,25(OH)_2_D_3_ in the hippocampus and prefrontal cortex.^(^ ^161^ ^)^

The antagonism between corticosterone and vitamin D would also appear to be reflected at a behavioral level. Chronic cortisol administration induces depression‐like phenotypes in animals; and in a remarkably consistent pattern, vitamin D would appear to ameliorate or completely reverse this. In terms of adult behavior, vitamin D reverses depressive behavioral phenotypes induced by chronic corticosterone.^(^ ^162^, ^163^, ^164^, ^165^ ^)^ Putative mechanisms include regulation of glucocorticoid receptor expression in hippocampus or via restoring DA levels in the reward centers of the brain.

DVD deficiency may also alter maternal stress responsivity in both rats ^(^ ^166^ ^)^ and mice.^(^ ^62^ ^)^ DVD deficiency also adversely affects maternal care,^(^ ^68^ ^)^ which, as previously outlined, can also produce long‐lasting changes in stress‐responsivity in offspring.^(^ ^67^ ^)^

## Conclusions

**Fig 1:**
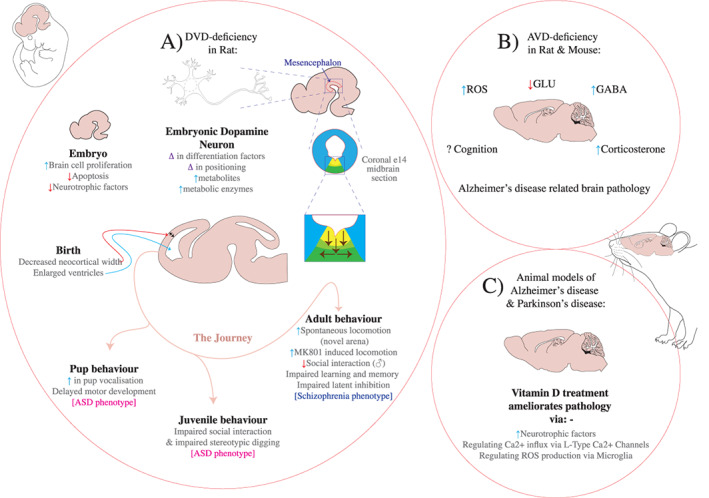
Vitamin D and its effects on brain and behavior. (*A*) Depicts the progressive molecular, cellular, brain structural and behavioral abnormalities induced in the developmental vitamin D (DVD)‐deficient rat model. (*B*) Far less investigation has been conducted on adult vitamin D (AVD)‐deficiency reflecting less certainty regarding the use of this model to study brain disorders. (*C*) There have been numerous studies in models of relevance to either Alzheimer or Parkinson disease indicating intervention with vitamin D may have therapeutic potential. Autism Spectrum Disorder (ASD) = _______; GABA = gamma‐aminobutyric acid; GLU = glutamate.

In this mini‐review, we have concentrated on summarizing the preclinical literature outlining vitamin D metabolism, and genomic and non‐genomic signaling in brain. We urge caution when interpreting previous constitutive KO strategies to genetically alter vitamin D signaling given the vast array of non‐brain–related alterations they induce. Similarly, the older dietary manipulations frequently induced hypercalcemia, which would obscure any individual contribution of vitamin D to brain function. In addition, there are also clear species and even strain differences with respect to the dietary effects of vitamin D deficiency in rodents. The current DVD‐ or AVD‐deficient rodent models have no such impediments producing animals that are normocalcemic and appear physiologically normal. The breadth of data obtained from such models confirms vitamin D as an important neurosteroid for both developing and adult brains, producing animals in which there are abnormalities in a diverse range of behavioral phenotypes of interest to both psychiatry and neurology. A summary of these findings is presented in Figure 1.

Epidemiological studies linking low neonatal vitamin D with disorders of brain development, such as autism and schizophrenia, continue to emerge. Similarly, the link between low levels of vitamin D and degenerative conditions such as AD and PD is progressively strengthening. As a result, speculation continues as to the relevance of adequate vitamin D levels in the possible prevention/amelioration of such disorders. This of course would be difficult to test directly for developmental conditions with an adult onset such as schizophrenia; therefore, this association can only be made retrospectively. However, for childhood‐onset psychiatric conditions and ongoing degenerative conditions, properly designed randomized clinical trials of vitamin D supplementation in such risk groups are likely to yield interpretable data in a timely fashion.

We again would like to insert a note of caution in light of certain recent high‐profile reports. Unfortunately, in many observational epidemiological studies of vitamin D status and psychiatric outcomes, the issue of reverse causality (the condition induces low levels of vitamin D rather than the reverse) is often not, or is poorly addressed. This is made all the more relevant give a very high‐profile recent report in the *New England Journal of Medicine* showing virtually all mental illnesses were associated with an increased risk of a subsequent nonpsychiatric medical condition.^(^ ^167^ ^)^ This has significant implications for psychiatric research in general. It is also highly relevant to any proposed association between low vitamin D levels reported in adults with any psychiatric or neurological condition because as sick people they are probably not looking after their diet or getting adequate exercise and exposure to sunshine. This is of perhaps diminished relevance to conditions associated with DVD deficiency. We urge all future epidemiological studies that seek to examine the relationship between vitamin D and psychiatric or neurological conditions to rigorously control for the established poor general health of patients with psychiatric conditions.

It is also salient to mention another recent landmark study that used Mendelian randomization models to examine gene pathways related to 25(OH)D_3_ blood concentrations. This study could find no evidence that genetic factors involved in the production of 25(OH)D_3_ were causal for psychiatric disorders.^(^ ^168^ ^)^ To us, this suggests any link between 25(OH)D_3_ levels and any brain‐related outcome are likely to be solely driven by environmental factors.

By now, there has been sufficient interest in the links between vitamin D and brain‐related disorders for contrary findings to begin to emerge. For instance, it is illustrative to examine a number of recent publications showing a predicted inverse relationship between DVD deficiency and autism.^(^ ^10^, ^11^, ^12^, ^169^ ^)^ These studies all had mean 25(OH)D_3_ levels of <50nM, which is considered by some authors to represent a cutoff for vitamin D deficiency. Two recent studies have failed to find this inverse association.^(^ ^170^, ^171^ ^)^ So, at face value, this appears a failure to replicate previous studies. However, it is crucial to note that in these last two studies, the mean levels of 25(OH)D_3_ were actually very high (>70 to 80nM), and there were very few individuals that were actually vitamin D deficient—meaning the association could not be properly addressed. These same six studies all used the same laboratory to analyze samples, so technical bias (so common among vitamin D studies in different populations) could be ruled out. This suggests a threshold effect rather than any continuous relationship between DVD deficiency and autism. We highlight this particular relationship to illustrate some of the confusion regarding statements regarding potential causality between vitamin D and various brain‐related clinical disorders.

We believe that if all such possible confounds can be carefully considered in the future, then more clarity might be brought to the next generation of epidemiological investigations examining vitamin D levels in psychiatric or neurological conditions. It remains an extremely attractive option to use such a simple, safe, and inexpensive intervention to alleviate the substantial disease burden these conditions carry for patients. Given the alarming prevalence of hypovitaminosis D in both pregnant women and their newborns^(^ ^172^ ^)^ and in the general population, ensuring the diverse functional capacities of this neuroactive steroid in the developing and adult brain are preserved through either environmental or dietary interventions would appear to be a vital public health priority. Ultimately, only well‐designed randomized double‐blinded clinical trials will reveal the therapeutic relevance of vitamin D in brain‐related disorders.

## Disclosures

The author has no conflicts of interest.

### PEER REVIEW

The peer review history for this article is available at https://publons.com/publon/10.1002/jbm4.10419.
